# Accurate reconstruction of viral genomes in human cells from short reads using iterative refinement

**DOI:** 10.1186/s12864-022-08649-8

**Published:** 2022-06-06

**Authors:** Sau-Dan Lee, Man Wu, Kwok-Wai Lo, Kevin Y. Yip

**Affiliations:** 1 Department of Computer Science and Engineering, The Chinese University of Hong Kong, Shatin, Hong Kong; 2 Department of Anatomical and Cellular Pathology, The Chinese University of Hong Kong, Shatin, Hong Kong; 3 Current address: Sanford Burnham Prebys Medical Discovery Institute, La Jolla, 92037 CA USA

**Keywords:** Viral genomes, Epstein-Barr Virus, Cancer, Whole-genome sequencing, Iterative refinement

## Abstract

**Background:**

After an infection, human cells may contain viral genomes in the form of episomes or integrated DNA. Comparing the genomic sequences of different strains of a virus in human cells can often provide useful insights into its behaviour, activity and pathology, and may help develop methods for disease prevention and treatment. To support such comparative analyses, the viral genomes need to be accurately reconstructed from a large number of samples. Previous efforts either rely on customized experimental protocols or require high similarity between the sequenced genomes and a reference, both of which limit the general applicability of these approaches. In this study, we propose a pipeline, named ASPIRE, for reconstructing viral genomes accurately from short reads data of human samples, which are increasingly available from genome projects and personal genomics. ASPIRE contains a basic part that involves de novo assembly, tiling and gap filling, and additional components for iterative refinement, sequence corrections and wrapping.

**Results:**

Evaluated by the alignment quality of sequencing reads to the reconstructed genomes, these additional components improve the assembly quality in general, and in some particular samples quite substantially, especially when the sequenced genome is significantly different from the reference. We use ASPIRE to reconstruct the genomes of Epstein Barr Virus (EBV) from the whole-genome sequencing data of 61 nasopharyngeal carcinoma (NPC) samples and provide these sequences as a resource for EBV research.

**Conclusions:**

ASPIRE improves the quality of the reconstructed EBV genomes in published studies and outperforms TRACESPipe in some samples considered.

**Supplementary Information:**

The online version contains supplementary material available at (10.1186/s12864-022-08649-8).

## Background

According to the GLOBOCAN 2018 database, an estimated 9.9% of new cancer cases are linked to infection of DNA viruses [1, 2]. Approximately 1.6 million DNA virus-associated cancers per year were reported worldwide. The major tumor DNA viruses involved include human papillomavirus (HPV), hepatitis B virus (HBV), and EBV, which are associated with cervical cancer, liver cancer, and NPC, respectively, and other cancers. Identifying these pathogens is important for understanding cancer development and has led to the successful development of vaccines for reducing cancer risks and identification of specific biomarkers for improving clinical management.

In the case of NPC, almost all tumors harbor clonal EBV genomes in episomal form [3]. Indeed, persistent EBV latent infection in nasopharyngeal epithelial cells is a critical early event in NPC tumorigenesis. Intriguingly, although 90% of people worldwide are with asymptomatic lifelong EBV infection, global NPC incidence is less than one per 100 000 persons per year [4]. In addition, this EBV-associated cancer is endemic and most prevalent in Southeast Asia and southern China, with the incidence up to 24 per 100 000 persons. It is thus tempting to suspect that some particular EBV strains are partially associated with NPC, in addition to other complex genetic and environmental factors. Supporting this, a recent study on EBV strains from southern China has identified two non-synonymous mutations within the BALF2 gene that are strongly associated with NPC risks (with odds ratios of 8.69 and 6.14, respectively) [5]. The prevalence of these strains may contribute to the exceptionally high rate of NPC in southern China. In general, the genetic variations specific to tumor-associated EBV strains may provide important insights into the development of vaccines and therapeutics [6]. Yet, the exact EBV strains that are linked to the pathogenesis of NPC and the underlying cellular and molecular mechanisms remain not fully understood [7].

A major obstacle to the identification of cancer-specific viral strains is the limited number of reliably reconstructed viral genome sequences in human cells [3]. Realizing the importance of these viral genome sequences, some studies have used a variety of experimental and computational methods to deduce them from cancer and normal samples [3, 5–8]. Experimentally, the common methods include amplicon sequencing, target capture followed by sequencing, and whole-genome sequencing (WGS), which have different levels of enrichment of sequencing reads coming from the viral genome as compared to the host genome. Computationally, the viral genome has been reconstructed from the sequencing reads using reference alignment, de novo assembly, or a combination of both. The reconstructed sequences are sometimes refined, with errors corrected and gaps filled, by supplementary sequencing of specific regions.

We argue that there are several key advantages of reconstructing viral sequences from WGS data based on short-read sequencing alone. First, as compared to amplicon-based and capture-based methods, which require protocols specially designed for the target viruses, WGS produces sequencing reads from viruses in the host cells using standard sequencing protocols. Second, the resulting data of WGS can detect multiple types of virus at the same time, including those in episomes and those integrated into the host genome. Third, and most importantly, WGS data of human samples have been increasingly available at an astonishing rate due to various large-scale genome projects from particular populations and research consortia [9–11]. The sample sizes involved in these projects are unmatched by typical research projects, especially in terms of the number of healthy controls. Sequencing data produced by personal genomics companies represent another rich source of data for reconstructing the associated viral sequences, which is not commonly done in most standard analysis pipelines currently. Having an effective computational method for reconstructing viral genomes from these data can thus facilitate the generation of additional information about the sequenced individuals at no extra experimental costs.

Accurate reconstruction of viral sequences from WGS data is not without challenges. Alignment-based methods rely on the availability of a suitable reference genome. Fast-evolving viruses could have their genomic sequences differing from the reference in major ways, involving not only small variations but also large structural variations. Methods based on de novo assembly, on the other hand, face difficulties related to non-unique sequences, incomplete genome coverage, sequencing errors and sequence heterogeneity. For instance, the EBV genome contains an IR1 repeat region of around 23 kbp in length. The exact length of this region can hardly be deduced accurately in a de novo assembly. The mixture of reads from both the viral and host genomes, possibly together with other genomes contained in the samples such as other pathogens, further complicates the situation. Third-generation sequencing methods can produce long reads that help overcome some of these issues. However, in addition to their much higher error rates as compared to short-read sequencing [12], their high cost and low adoption by major genome projects thus far also limit their use in large-scale investigations of disease-associated viral genomes. In this paper, we propose a new pipeline, ASPIRE (ASsembly Pipeline with Iterative REfinement), for accurately reconstructing viral genomes from human WGS data. It combines the advantages of de novo assembly and sequence alignment to resolve different types of issues using an iterative refinement procedure. Applying ASPIRE to the WGS data of both our in-house NPC samples and NPC samples from previous studies, we show that the EBV genomes reconstructed by our pipeline is more accurate than those produced by other pipelines as evaluated by the rate and quality of read alignments.

## Implementation

### The viral genome reconstruction method

We developed a new method, ASPIRE, for reconstructing viral genomes in host cells from WGS data. It consists of a basic pipeline and a novel iterative refinement procedure that is essential to the accuracy and completeness of the reconstructed viral genomes.

Before entering into the basic pipeline, the sequencing reads are first aligned to the reference sequence of the host genome. The aligned reads are filtered and the remaining ones are collected to form a set of potential reads coming from the viral genome. Additional rounds of filtering can also be performed to remove sequencing reads from other unwanted sources of DNA. For example, if the WGS data are produced from a human-in-mouse xenograft, the reads can be additionally filtered against the mouse genome.

**Fig. 1:**
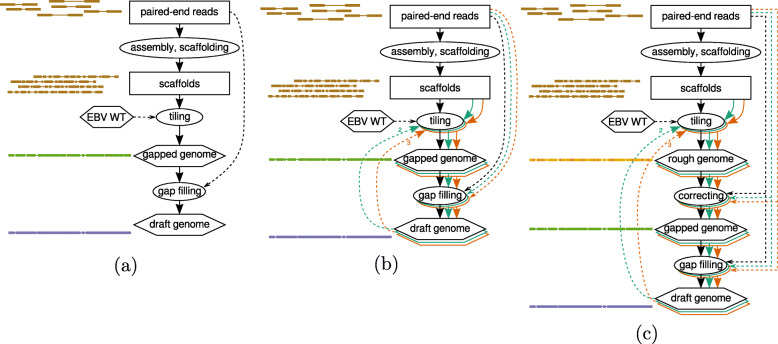
The ASPIRE method for reconstructing viral genomes. The basic pipeline (**a**) includes contig assembly, scaffolding, tiling, and gap filling. The iterative refinement procedure involves repeated alignment of scaffolds to the latest version of the reconstructed viral genome followed by gap filling (**b**) and a correction step based on allele frequencies derived from read alignments (**c**)

The remaining reads then go through the basic pipeline (Fig. 1a). First, a de novo assembly of the reads is performed by SPAdes [13] to produce the contigs and subsequently the scaffolds. Contig polishing can be performed before scaffolding if necessary. Next, the scaffolds are aligned to a reference sequence of the viral genome using MUMmer 3 [14] to filter out scaffolds unlikely to belong to the viral genome and to order and orient those that do. The output of this “tiling” step is a gapped genome. Finally, the original set of filtered reads is used to perform gap filling using GapFiller [15].

The quality of the reconstructed genome produced by the basic pipeline depends heavily on the similarity between the reference viral genome and the viral genome in the sequenced sample. In particular, in the tiling step, some scaffolds may not be aligned to the reference if the sequences involved are not sufficiently similar. After gap filling, it is possible for the reconstructed genome to contain more information for aligning these scaffolds. Therefore, it could be useful to perform the tiling step again by aligning the scaffolds to the reconstructed genome, followed by another round of gap filling. This process can be repeated for multiple times to achieve iterative refinement (Fig. 1b).

Sequencing errors could also affect the quality of the reconstructed genome. During the tiling step, scaffolds are aligned without referring to allele frequencies, and this is common to many existing tiling tools. Therefore, even if the majority of sequencing reads support an alternative allele and only a few reads support the reference allele due to sequencing errors, the reconstructed genome may still prefer the reference allele since the corresponding contig aligns better to the reference. To deal with this problem, ASPIRE also includes a correction step that aligns the sequencing reads to the tiled rough genome (Fig. 1c). Based on the alignment results, sequence variants are called and the rough genome is updated with the most frequent alleles, before the updated sequence is supplied to the gap filling step.

### Technical details of the implementation

ASPIRE is available [16] as a command-line tool with subcommands to perform various steps (Fig. 1c, Fig. S1). Some technical details are explained below.

#### Assembly and scaffolding

De novo contig assembly and scaffolding are performed at once as shown in Fig. 1 with SPAdes version 3.10.1 [13] with the –careful option on the pre-processed WGS reads to generate the scaffolds for the subsequent steps. In the experiments in “Using an alternative de novo assembler” (Fig. 8), ASPIRE was run with the “–sga” option to the subcommand “assemble”, which causes SGA version 0.10.15 [17], instead of SPAdes, to be employed for assembly and scaffolding.

#### Tiling

The scaffolds are filtered and pieced together using the MUMmer 3 suite [14]. Three tools from this suite are run with the options shown below, in this order:

1. nucmer –maxmatch -b 4000 -c 30
2. delta-filter -q
3. show-tiling -c -R

For Step 1, a reference genome is needed as input. In our application of ASPIRE to reconstruct EBV genomes from NPC samples, the EBV wild-type genome (NC_007605.1) [18] was used as the reference in the basic pipeline (Fig. 1a) and in the first iteration of the iterative pipeline and corrective pipeline (Fig. 1b, c). For subsequent iterations, the reconstructed genome of the gap-filling step of the previous iteration was used as the new “reference”.

For NPC-16T, we needed to fine-tune the show-tiling tool with a parameter “-g 45000”. Otherwise, the resulting constructed genome would have a big deletion. This parameter made the scaffold filtering less stringent, causing the program to keep some big scaffolds that would otherwise be discarded. For the other samples, the default value (-g 1000) worked fine.

#### Correction

The correction step takes the rough genome created in the tiling step (see Fig. 1c) and corrects it according to the original WGS reads, with the aim of making the former more consistent with the latter.

In particular, we first use Bowtie2 [19] (with default settings) to align the WGS reads to the rough genome. Next, samtools [20] (“samtools mpileup”, or “bcftools mpileup” [21] in more recent versions) are used to call SNVs and indels. The commands and options used are:

1. samtools mpileup –redo-BAQ –max-idepth 1000000 –max-depth 1000000 –min-MQ 20 –BCF –output-MQ –output-tags=INFO/AD,INFO/ADF,INFO/ADR,DP,SP
2. bcftools call –multiallelic-caller –variants-only
3. bcftools filter -e ’ALT=~.~ || QUAL<20 || AD[0]+AD[1]<100’

The resulting SNV and indel list are used to correct the rough genome, using “bcftools consensus -e ’AD[0] >= AD[1]”. This produces a corrected genome.

The above procedure is repeated until no more significant improvements (≥0.3) in the lower quartile of MAPQ values can be achieved during the alignment.

#### Gap filling

The gapped genome (see Fig. 1) is fed to GapFiller [15] version 1.10 for gap filling. It is run with options “-t 0 -m 75 -o 5 -r 0.6 -i 50” with library parameters “FRAGMENT_SIZE=450”, “FRAGMENT_SIZE_ERROR=0.25”. The output is the result of the draft genome at the end of each iteration.

### Application of the basic pipeline to NPC data

We first applied the basic pipeline (Fig. 1a) to the WGS data of 61 NPC primary tumor samples to reconstruct their EBV genomes [22]. The NPC samples and the WGS experiments have been described before [22]. Briefly, all 61 NPC tumor tissues were collected by endoscopy or surgery, embedded in optimal cutting temperature (OCT) compound and stored at −70 ^∘^C. DNA samples extracted from microdissected frozen tissue sections were subjected to WGS, performed on Illumina HiSeq 2000 or HiSeq X platform with the TruSeq Nano DNA library preparation kit. The first 27 samples (NPC-1T to NPC-27T) were sequenced at a coverage of 60 ×, giving paired end reads of length 100 bp each. The remaining 34 samples (NPC-28T to NPC-51T, NPC-53T to NPC-62T) were sequenced at a coverage of 90 ×, giving paired end reads of length 150 bp each.

EBV is a double-stranded DNA virus with a circular genome containing 171 823 bp in the wild-type reference sequence [18]. In the pre-filtering step, the WGS sequencing reads were aligned to the human reference genome hg19 using Isaac [23] versions 01.14.03.12, 01.14.07.17 and 01.15.02.08. For EBV genome assembly, read pairs with one or both ends mapped to the human genome were discarded. The remaining reads were aligned to the mouse reference genome mm10 using Bowtie2 [19] with default parameters. Again, read pairs with one or both ends mapped were discarded. This left us with around 1.5% of the original reads for reconstructing the EBV genomes (Table S1).

For the particular data sets we considered, we obtained the alignment files directly from the sequencing center. To make sure that there were no residual adapters going into the subsequent steps of ASPIRE, we performed adapter trimming again. Adapter sequences used during sequencing were identified with FastQC version 0.10.1. They were trimmed from the reads using cutadapt version 1.13 [24], with parameters “-a AGATCGGAAGAGCACACGTCTGAACTCCAGTCAC -A AGATCGGAAGAGCGTCGT GTAGGGAAAGAGTGTAGATC TCGGTGGTCGCCGTATCATT –cut 6 -U 6 -q 20–trim-n –minimum-length 70 –discard-trimmed”. Reads that became shorter than 70 bp after trimming were discarded. The resulting DNA reads were then used for our assembly of EBV genomes.

**Fig. 2:**
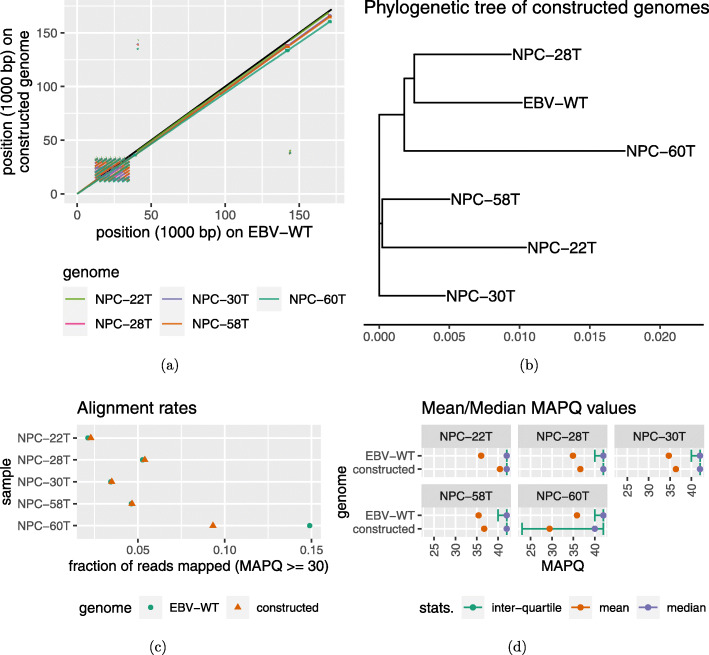
Comparing the wild-type EBV genome and the reconstructed viral genomes in five random samples by the basic pipeline. Whole-genome alignment (**a**) and phylogenetic tree analysis (**b**) show that the reconstructed genomes are similar to the wild-type, but the number of sequencing reads with high MAPQ is low in NPC-60T when they were aligned to the reconstructed genome, both relative to the total number of filtered, trimmed reads (**c**) and the number of aligned reads (**d**)

Figure 2a shows the alignments of the reconstructed viral genomes in five randomly chosen samples with the wild-type EBV genome. Except for the IR1 repeat region 12–35 kbp), the different parts of the reconstructed genomes were aligned sequentially with the wild-type, indicating that they differed from the wild type only by small variants. This high similarity between the reconstructed genomes and the wild-type was reinforced by a phylogenetic tree analysis (Fig. 2b), which showed that the wild-type sequence mixed well with the reconstructed sequences.

Specifically, the phylogenetic trees were generated as follows. First, the collection of genomes was used as input to Spaced [25], which was run with options “-r -d EV”, to produce an inter-genome distance matrix. Then, the nj function (neighbor-joining tree estimation) in the R package ape was invoked to derive a phylogenetic tree from the distance matrix. Finally, the tree was plotted with the ggtree package in R.

While it was encouraging that the reconstructed genomes were similar to the wild-type, the results above could not provide an unbiased evaluation of the quality of these reconstructed genomes because the wild-type genome was directly involved in the tiling step. We reasoned that if a reconstructed genome is of high quality, the sequencing reads should align to it as well as, if not better than, to the wild-type genome. To see if this was the case, we examined the number of reads with a mapping quality (MAPQ) ≥ 30, which corresponds to an alignment error probability ≤ 0.001. As expected, for four of the samples (NPC-22T, NPC-28T, NPC-30T and NPC-58T), the number was slightly higher when the reads were aligned to the reconstructed genome than to the wild-type; Yet for one sample (NPC-60T), the former was substantially smaller than the latter (Fig. 2c). Upon checking all the aligned reads in this sample, we also found that many of them had low mapping quality (Fig. 2d). Therefore, both the absolute number of high-quality alignments and the ratio of high-quality alignments among all alignments suggest that the basic pipeline failed to reconstruct the viral genome in this sample accurately. When we checked all 61 samples, we found that this problem happened to four samples (NPC-33T, NPC-42T, NPC-46T and NPC-60T).

### Application of iterative refinement to the NPC data

**Fig. 3:**
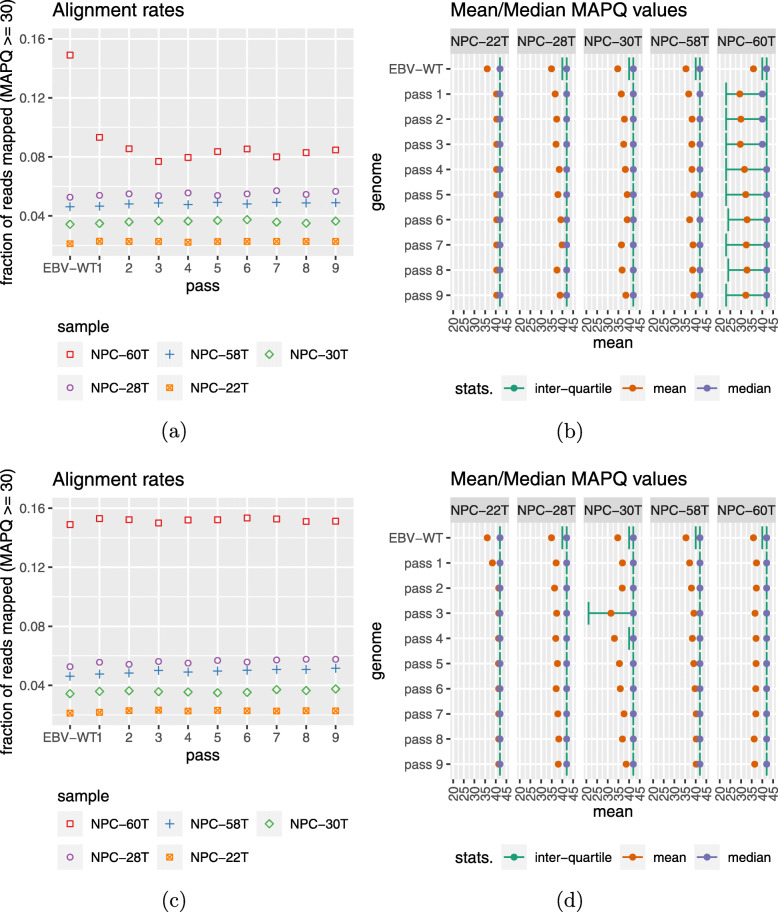
Comparing the wild-type EBV genome and the reconstructed viral genomes in the five random samples with iterative refinement. The results involving only the repeated tiling and gap filling steps (**a** and **b**) indicate that they could not address all the issues, while the error correction step (**c** and **d**) provided substantial further improvements to some samples

When we applied iterative refinement without the correction step (Fig. 1b) to the four “good” samples mentioned above, both the number of reads with MAPQ ≥ 30 (Fig. 3a) and the distribution of MAPQ values (Fig. 3b) improved when the reads were aligned to the reconstructed genomes. This indicates that the reconstructed genomes became more and more consistent with the sequencing reads, which suggested that the quality of the reconstructed genomes improved, although it is not a direct proof of correctness. For some of these samples, the improvement stopped after one iteration (e.g., NPC-22T), while for some other samples, the improvement continued for many iterations (e.g., NPC-28T). However, the reconstructed genome of the “bad” sample (NPC-60T) was not improved by the iterative re-alignments alone. We therefore also applied the correction step to the iterative refinement procedure (Fig. 1c). This correction step successfully improved both the number of reads with high MAPQ (Fig. 3c) and the distribution of MAPQ values (Fig. 3d). Most importantly, for all five samples, read alignments to the reconstructed genomes were now better than to the wild-type genome in terms of both measures.

The number of iterations in the iterative refinement procedure required was sample-specific. In the five samples, two iterations seemed to be sufficient to achieve most of the improvements. Based on this, we applied ASPIRE to all 61 samples with two iterations of refinement, and found that read alignments were improved in general (Fig. S2). In particular, the mean MAPQ was higher in all 61 cases when reads were aligned to the reconstructed genomes than when they were aligned to the wild-type (Fig. S2b).

### Wrapping for circular genomes

**Fig. 4:**
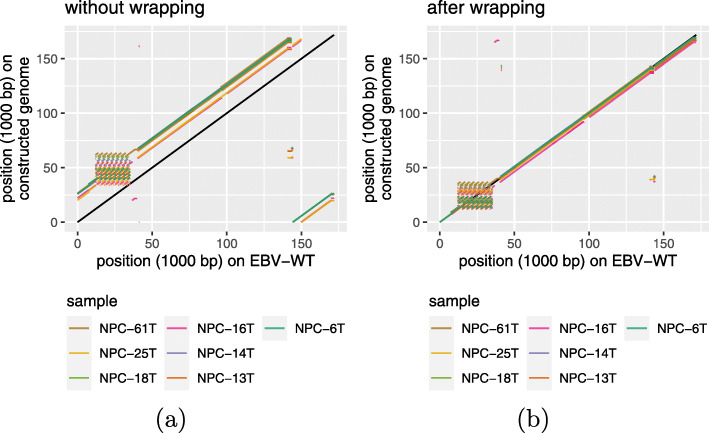
Alignments between the wild-type EBV genome and the reconstructed genomes in seven samples with the rotation issue. The rotated regions are clearly seen before applying the wrapping step (**a**), and they are resolved after applying the step (**b**)

For viruses with a circular genome such as EBV, the starting point of the linear representation of the reference genome is usually defined manually using information not available to the de novo assembly method. For example, the linear representation of the EBV genome has a starting point in the terminal repeat region within intron 1 of the LMP-2B gene, but during the de novo assembly step, some contigs may span this point. Correspondingly, the reconstructed linear genome could be “rotated”, with a region at the end of the linear representation moved to the beginning, or vice versa. We found this happened in seven of our 61 samples (Fig. 4a). To deal with this issue, we augmented ASPIRE with a final wrapping step such that the reconstructed genome could be linearly aligned to the wild-type genome. This additional step successfully solved the issue in all seven samples (Fig. 4b).

### The TRACESPipe pipeline

In order to evaluate the performance of ASPIRE, we also compared it with another pipeline, TRACESPipe [26]. TRACESPipe version 1.0.1 was downloaded and input files were prepared as described in the README.md file. Following the instructions in section 5.1 of README.md, the following command was issued to run the pipeline:


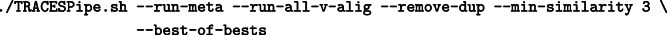


FASTA files for the constructed genomes were found under +output_data/TRACES_viral_consensus/+.

## Results

### The final reconstructed EBV genomes

**Fig. 5:**
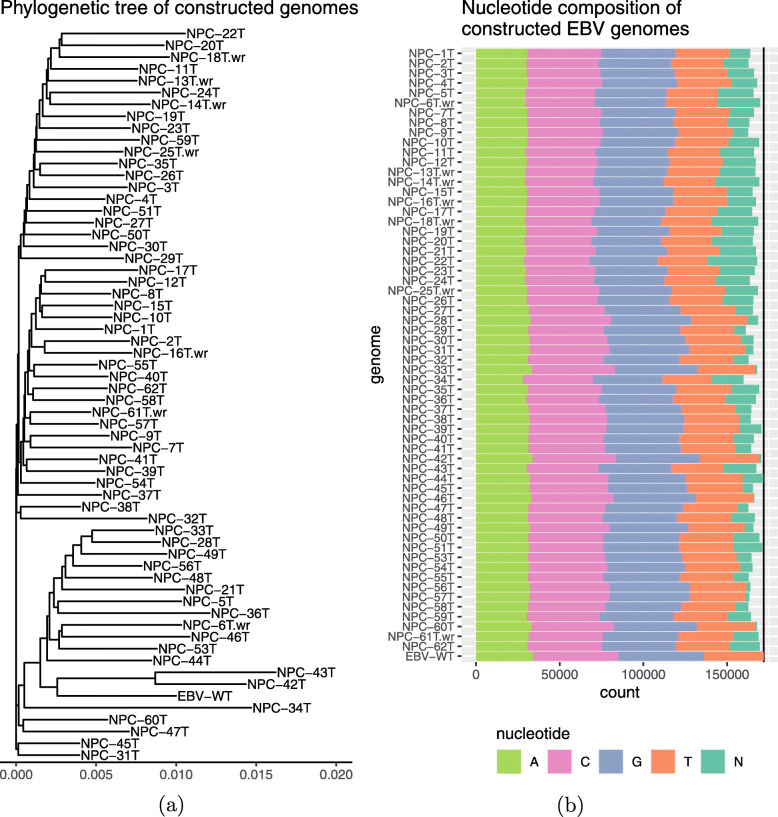
Comparing the EBV wild-type genome with the 61 reconstructed genomes from the NPC samples. These genomes are compared by their phylogenetic relationships (**a**) and nucleotide compositions (**b**). In both panels, the wild-type is marked as “EBV-WT”, while the reconstructed genomes that required the wrapping step have the suffix “.wr” in their labels

We applied the complete ASPIRE pipeline [16] with two iterations of refinement to all 61 NPC samples and the wrapping step to the seven samples. All these reconstructed sequences have been made accessible at European Nucleotide Archive (BioProject ID PRJEB42525). Figure 5a shows the phylogenetic tree involving the 61 reconstructed EBV genomes in these samples and the wild-type genome. The tree contains three main branches, with the wild-type genome (EBV-WT) belonging to one of them far away from the other two, indicating that many of the reconstructed EBV genomes were closer to each other than to the wild-type. This is consistent with the origins of the samples. Specifically, the wild-type EBV genome was assembled from data of the B95-8 and Raji strains, where B95-8 is a cell line established by infecting marmoset lymphocytes with a virus of American infectious mononucleosis origin [27, 28], while Raji is a cell line derived from a Nigerian patient with Burkitt’s lymphoma [29]. In contrast, the samples included in the current study were all from NPC patients of Asian origin. Due to the different diseases and ethnicities of origin involved, the EBV strains in our NPC samples are expected to have various systematic differences from the wild-type. One of these differences observed is the genome size, with most of the reconstructed genomes shorter than the wild-type (Fig. 5b), which also suggests that the higher alignment rate of the reads to the reconstructed genomes than to the reference was not due to the inclusion of non-EBV sequences to these reconstructed genomes.

### Comparison with other pipelines

#### Our dataset

**Fig. 6:**
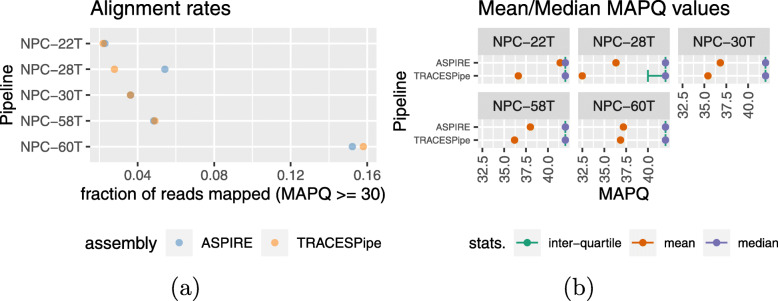
Comparing the genomes constructed by ASPIRE and TRACESPipe. While the alignment rates (**a**) are comparable, ASPIRE excels in mapping quality (MAPQ) scores (**b**). It has much higher mean MAPQ, and the interquartile range is narrower

Previously, another pipeline called TRACESPipe has been proposed for automatic and efficient identification, assembly, and analysis of viral genomes [26]. It relies on cooperation among 3 modalities: compression-based prediction, sequence alignment, and de novo assembly. However, unlike ASPIRE, it does not incorporate iterative refinement. We tested it on our five randomly chosen NPC samples and found that although the alignment rates (for MAPQ≥30) were similar when aligning reads to the genomes reconstructed by ASPIRE and TRACESpipe, the alignment quality, in terms of MAPQ scores, was much higher for the ASPIRE genomes (Fig. 6). These results suggest that the iterative refinement step is crucial for ensuring the accuracy of the reconstructed viral genomes.

#### More datasets

To demonstrate the utility of ASPIRE, we further applied it to data from two other studies of EBV genomes in NPC samples [5, 7]. In Hui et al. [7], EBV genomes were reconstructed from NPC WGS data by a de novo sequence assembly followed by aligning the assembly results directly to the wild-type genome and linearizing the resulting sequence based on the wild-type configuration. Sequencing reads were then aligned to the reconstructed genomes, and nucleotides without sufficient supports from high-quality alignments were marked as ambiguous (‘N’). Xu et al. [5] instead used a capture-based protocol to produce sequencing reads enriched for EBV sequences, followed by aligning the reads directly to the wild-type genome without performing any de novo assembly. For each of these two studies, we randomly selected three NPC samples (Table 1), downloaded their sequencing data, and applied our full pipeline (Fig. 1c) with two passes of iterative refinement including the correction step.

**Table 1:** Six samples randomly selected from two other studies for comparing with ASPIRE

Publication	Sample	ENA accession
[7]	HKHD92	MH590461.1
[7]	HKHD98	MH590467.1
[7]	HKHD113	MH590482.1
[5]	NPCT014	MK540372.1
[5]	NPCT094	MK540451.1
[5]	NPCS009	MK540321.1

**Fig. 7:**
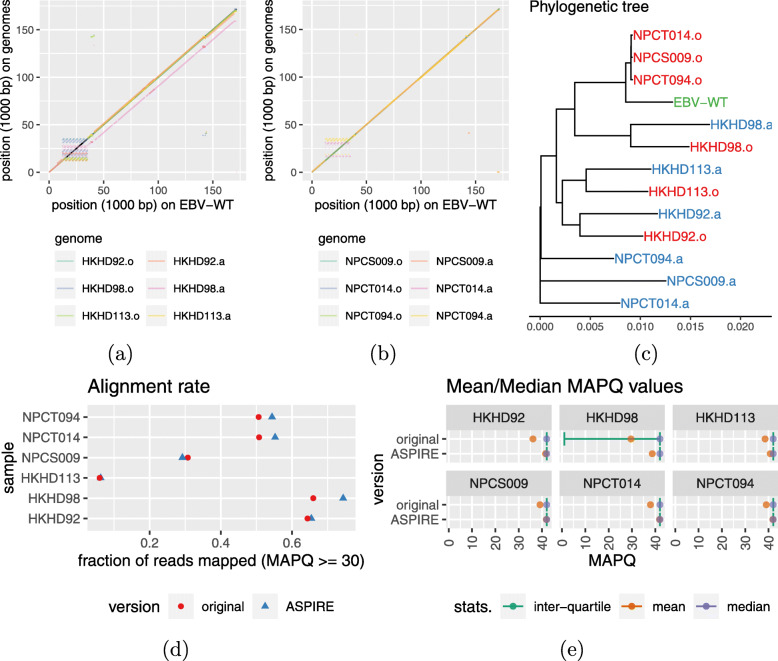
Comparing the six EBV genomes obtained from two other studies with the ones we reconstructed from their sequencing data. The alignments of these genomes with the wild-type genome (**a** and **b**) and the phylogenetic tree of all the sequences (**c**) both show differences between the original genomes and the ones reconstructed by us. In these panels, genomes obtained from the original studies and those reconstructed with ASPIRE have suffix “.o” and “.a” in their labels, respectively. The two versions are also compared by the number of reads with an MAPQ ≥ 30 (**d**) and their distributions of MAPQ values (**e**)

As a first exploration, we aligned both the EBV genomes obtained from the original studies and the ones we reconstructed with the wild-type (Fig. 7a, b). Most of these genomes were highly similar to the wild-type except for our reconstructed genome from the sample HKHD98, which was around 10 kbp shorter mainly in the IR1 repeat region. To more systematically compared these genomes, we produced a phylogenetic tree based on their sequences. Interestingly, we found that for the three samples from Hui et al. [7], each of the reconstructed genomes was grouped with the corresponding genome from the original study; In contrast, for the three samples from Xu et al. [5], our reconstructed genomes all grouped together while the ones from the original study formed a separate group together with the wild-type sequence. Together, these two sets of results show that our reconstructed genomes differed from the ones from either of the two studies.

We then aligned the sequencing reads to the reconstructed genomes. Among the six samples, three of them had clearly more reads with MAPQ ≥ 30 when the reads were aligned to our reconstructed genomes than to the ones from the original studies, while two others also had slight improvements (Fig. 7d). Our reconstructed genomes also led to larger mean MAPQ values of the aligned reads in all six samples (Fig. 7e). The improvement was most substantial for the sample HKHD98, of which our method inferred a 10 kbp deletion as compared to the wild-type. These results suggest that ASPIRE was useful for determining the differences between the wild-type and the genomes to be reconstructed.

### Testing on cells with a low EBV copy number

The EBV copy number in most EBV-infected B cells and EBV-positive lymphoma cell lines ranges from 2 to >50 [30]. In some of the NPC samples considered in this study, the EBV copy number was relatively high. To see whether the performance of ASPIRE depends on the EBV copy number, we also applied it to reconstruct the EBV genome sequences in NPC43 (5-6 copies of EBV genome per cell [31]) and C17 (2-3 copies of EBV genomes per cell [32]) cells. From the results, the reconstructed EBV genome sequences could be aligned well to the EBV reference genome (Fig. S3). The low EBV copy number in C17 cells did not appear to have created significantly more gaps or incorrect sequences in the reconstructed genome.

### Using an alternative de novo assembler

**Fig. 8:**
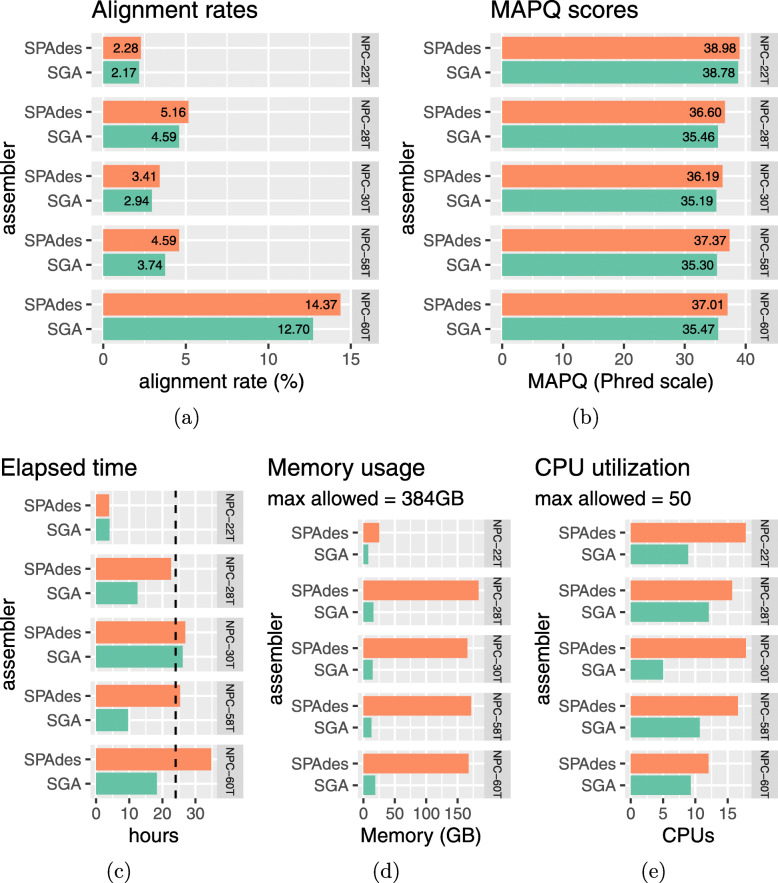
Comparison of running ASPIRE for 2 iterations with different de novo assemblers on five random samples. It is observed that using SGA for de novo assembly gives slightly lower alignment rates (**a**) and mapping qualities (**b**). However, the difference is relatively small. SGA, on the other hand, has the advantage of shorter execution times (**c**) and consuming much less memory (**d**) but it is less capable of CPU parallelism (**e**). The dashed vertical line in (**c**) indicates 24 hours

ASPIRE is a very modular pipeline, where each step in Fig. 1c is a data manipulation task that can be handled by difference tools. This allows us to replace and update any step with better tools in the future. As a demonstration, ASPIRE can optionally use SGA (String Graph Assembler) [17] for the assembly and scaffolding step, instead of SPAdes. We ran ASPIRE with SGA for 2 iterations for the five randomly selected samples as mentioned and compared the reconstructed genomes with ASPIRE using SPAdes as the assembler (see Fig. 8). We found that the resulting genomes using ASPIRE with SGA are slightly worse than those using ASPIRE with SPAdes in terms of alignment rates and mapping quality, but with SGA, the number of CPU cores required was smaller, the memory consumption was lower, and the running time was shorter. Therefore, the modular design of ASPIRE has allowed the user to tradeoff between the quality of the reconstructed genomes and reduction of computational resources.

## Discussion

In this work, we have developed an iterative assembly pipeline, ASPIRE, which starts by a de novo assembly of the reads and an alignment of the assembled scaffolds to a reference, followed by iterative refinements and corrections. We have used this pipeline to reconstruct the EBV genomes in 61 primary NPC tumor samples based on short read data from WGS. The reliability of the reconstructed genomes have been confirmed by having the sequencing reads aligned to them with higher quality as compared to aligning to the reference genome. By analyzing the situations of individual samples, we have shown the importance of each main step of our pipeline, including realignment of scaffolds to the reconstructed genome, correction of errors based on frequency of supporting reads, and wrapping of circular genome. The importance of iterative refinement cannot be emphasized enough. It enables ASPIRE to construct more accurate genomes than other pipelines, such as TRACESPipe and the ones used in two other studies [5, 7] in some of the samples considered.

ASPIRE is highly modular, with each step involving some well-defined tasks. While we have chosen some specific tools as the default for particular tasks, such as sequence assembly, scaffold alignment and gap filling, other tools can be easily plugged into our pipeline to replace these default options if they are considered more suitable based on the data properties. We have demonstrated this by running ASPIRE with an alternative de novo assembler, SGA, in the place of SPAdes (the default), and shown that this offers shorter finishing time at the expense of slightly reduced qualities of the constructed genomes. With such flexibility, both the effectiveness and efficiency of our pipeline can be improved from time to time by using improved tools for the individual tasks.

Although we have focused on WGS data in this work, ASPIRE can also be applied to other types of sequencing data as long as they contain sufficient information about the viral genome. For instance, we have demonstrated that our pipeline successfully reconstructed the EBV genomes in three randomly picked samples from Xu et al. [5], which used a capture-based protocol to enrich for EBV sequences instead of WGS. Of course, for sequencing methods that do not produce reads that cover the whole viral genome, such as whole-exome sequencing, it would be impossible to reconstruct the viral genome regardless of the computational pipeline.

The EBV genome that we focused on in this work is an episomal circular double-stranded DNA molecule when the virus is in its latent stage in NPC cells. For DNA viruses having a linear genome or those integrated into the host genome, ASPIRE should work equally well, potentially with the integration sites identified during the reconstruction process as a by-product. Some sequencing reads produced from regions around the integration sites, which appear as virus-human hybrid reads, could be accidentally filtered out in the initial alignment of sequencing reads to the human genome. Likewise, if some viral sequences have high homology to human sequences, they could also be unintentionally filtered out. In both cases, the reconstructed viral genomes would contain some gaps, and this issue could be partially dealt with by filling gaps using all sequencing reads rather than only those not aligned to the human genome.

For RNA viruses, if the number of copies per cell is comparable to the number of transcripts of genes with medium expression, our pipeline should also be able to reconstruct the viral genome from RNA sequencing data produced by a protocol that can pick up the viral sequences.

Since NPC is driven by clonal EBV infection, we did not expect the NPC samples we analyzed to harbor other herpesviruses. Similarly, other EBV-associated malignancies also have similar clonal features, and thus the consideration of additional herpesviruses in the reconstruction process is likely unnecessary. However, for samples that indeed contain multiple herpesviruses in the same cells, the viral genome reconstruction process could be affected, especially if the herpesviruses have highly similar sequences. This is a difficult situation not only for ASPIRE but for all methods that aim at reconstructing the viral genomes in general. In this situation, the sequence assembly process will likely detect substantial deviations from a simple linear sequence by having a lot of branching and apparent sequence variations. More advanced methods will be needed to infer the genome sequences of individual viruses.

In this work we have used real sequencing data to demonstrate the performance of ASPIRE. In the future, synthetic data with controlled properties can be produced to benchmark the performance of different methods for reconstructing viral genomes in human cells. It would also be useful to evaluate whether the performance of ASPIRE depends on the size of the viral genome using whole-genome sequencing data that contain other viruses of different genome sizes.

## Conclusions

By incorporating iterative refinement our modular pipeline, ASPIRE, improves the quality of the reconstructed EBV genomes reported in published studies and outperforms the TRACESPipe pipeline in some of the samples considered.

## Availability and requirements

**Project name:** ASPIRE (ASsembly Pipeline with Iterative REfinement)

**Project home page:** https://github.com/kevingroup/aspire

**Operating system(s):** Platform independent

**Programming language:** Perl 5

**Other requirements:** Perl 5.32.1, samtools 1.13, bcftools 1.13, cutadapt 3.4, SPAdes 3.13.1, SGA 0.10.15 (optional), MUMmer 3.23 or 4.x, Bowtie2 2.4.2, BWA 0.7.17, GapFiller 1-10, see https://github.com/kevingroup/aspire#prerequisitesfor details

**License:** GPL-3.0

**Any restrictions to use by non-academics:** see GPL-3.0

## Supplementary Information

**Additional file 1** Supplementary Figure S1. A more detailed view of the ASPIRE pipeline with the tools involved in every step shown.

**Additional file 2** Supplementary Figure S2. Comparing the wild-type EBV genome and the reconstructed viral genomes in all 61 samples with two iterations of refinement.

**Additional file 3** Supplementary Figure S3. Alignment of the EBV genomes reconstructed with ASPIRE in NPC43 and C17 cells to the reference EBV genome sequence.

**Additional file 4** Supplementary Table S1. Number of sequencing reads in each sample after different steps. The “raw”, “filtered”, “trimmed” and “EBV-WT” columns respectively show the number of millions of reads in the raw WGS data, after filtering human and mouse reads, after adapter trimming, and alignable to the EBV wild-type genome.

## Data Availability

ASPIRE is available [16] as a command-line tool to run the pipeline. The 61 EBV genomes constructed by ASPIRE from the WGS data of our NPC samples are accessible at European Nucleotide Archive (ENA) (BioProject ID PRJEB42525). Raw WGS alignments (in BAM format) are accessible at Europen Genome-phenome Archive (EGA) (Study ID EGAS00001004705). The three EBV genome sequences from Xu et al. [5] were obtained from NCBI GenBank (accession numbers MK540321.1, MK540372.1 and MK540451.1). Raw sequencing reads were downloaded from ENA (BioProject ID PRJNA522388). The three EBV genome sequences from Hui et al. [7] were obtained from NCBI GenBank (accession numbers MH590461.1, MH590467.1 and MH590482.1). Raw sequencing reads were downloaded from NCBI Sequence Read Archive (accession number SRP152584).

## Abbreviations

ASPIRE: ASembly Pipeline with Iterative REfinement
CPU: central processing unit
DNA: deoxyribonucleic acid
EBV: Epstein Barr virus
HBV: hepatitis B virus
HPV: human papillomavirus
MAPQ: mapping quality
NPC: nasopharyngeal carcinoma
RNA: ribonucleic acid
SGA: String Graph Assembler
WGS: whole-genome sequencing
