# Supplementary figures and images for: Accurate reconstruction of viral genomes in human cells from short reads using iterative refinement
Source: BMC Genomics. 2022 Jun 6;23:422. doi: 10.1186/s12864-022-08649-8 (PMC9169298; doi:10.1186/s12864-022-08649-8)

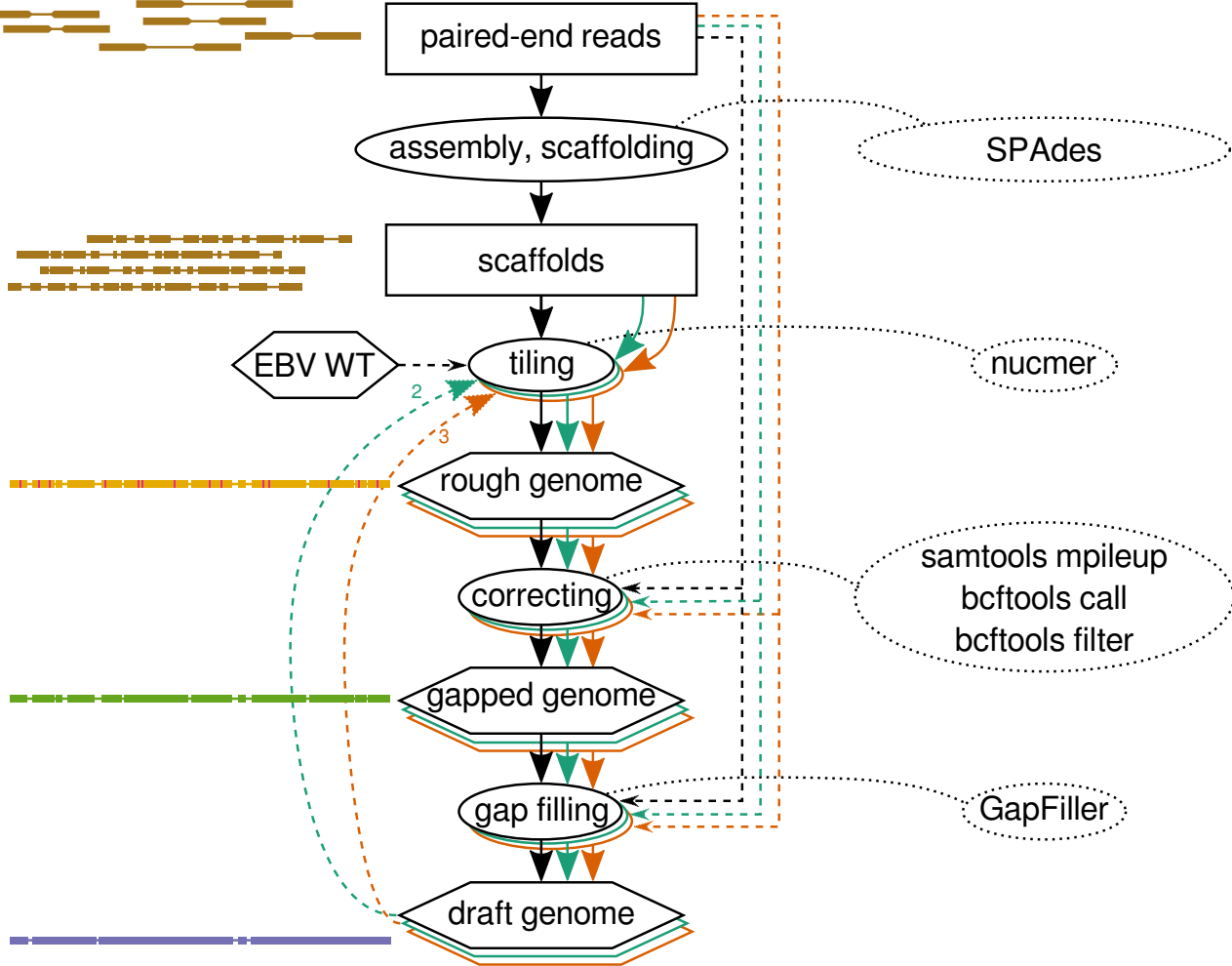

Supplement: Supplementary file 1 — Additional file 1 Supplementary Figure S1. A more detailed view of the ASPIRE pipeline with the tools involved in every step shown. [file 12864_2022_8649_MOESM1_ESM.pdf]

Alignment rates

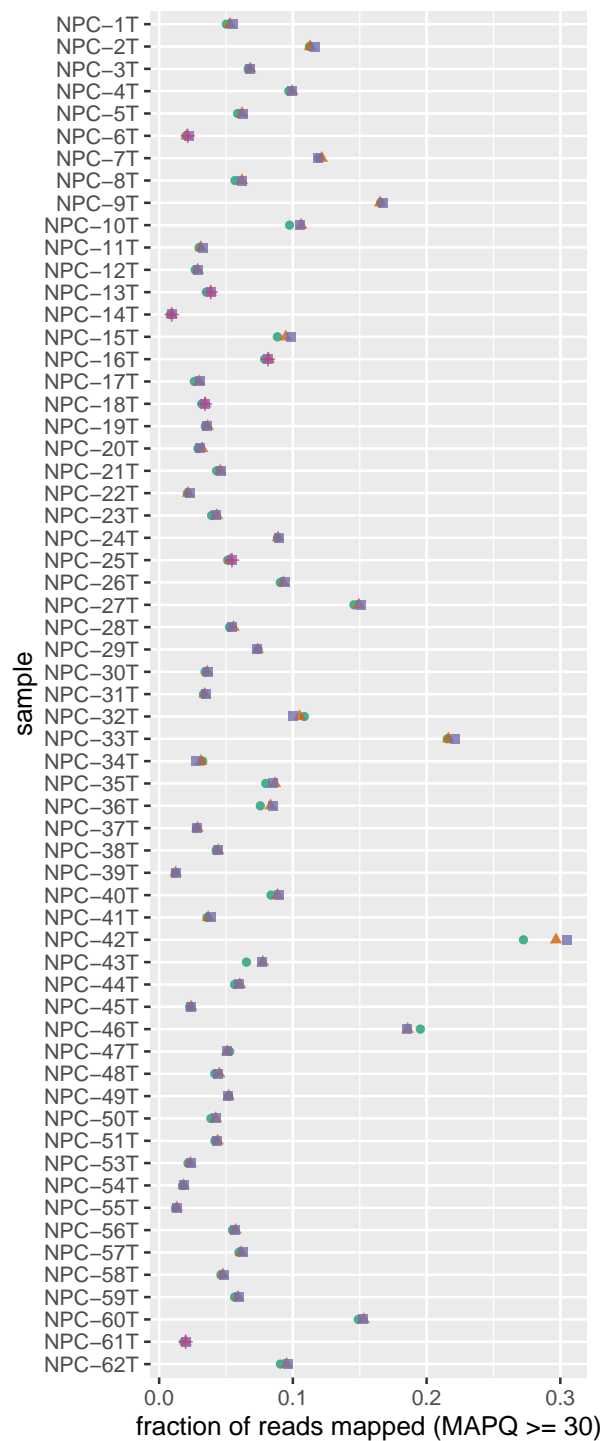

(a)

Mean/Median MAPQ values

(2 passes)

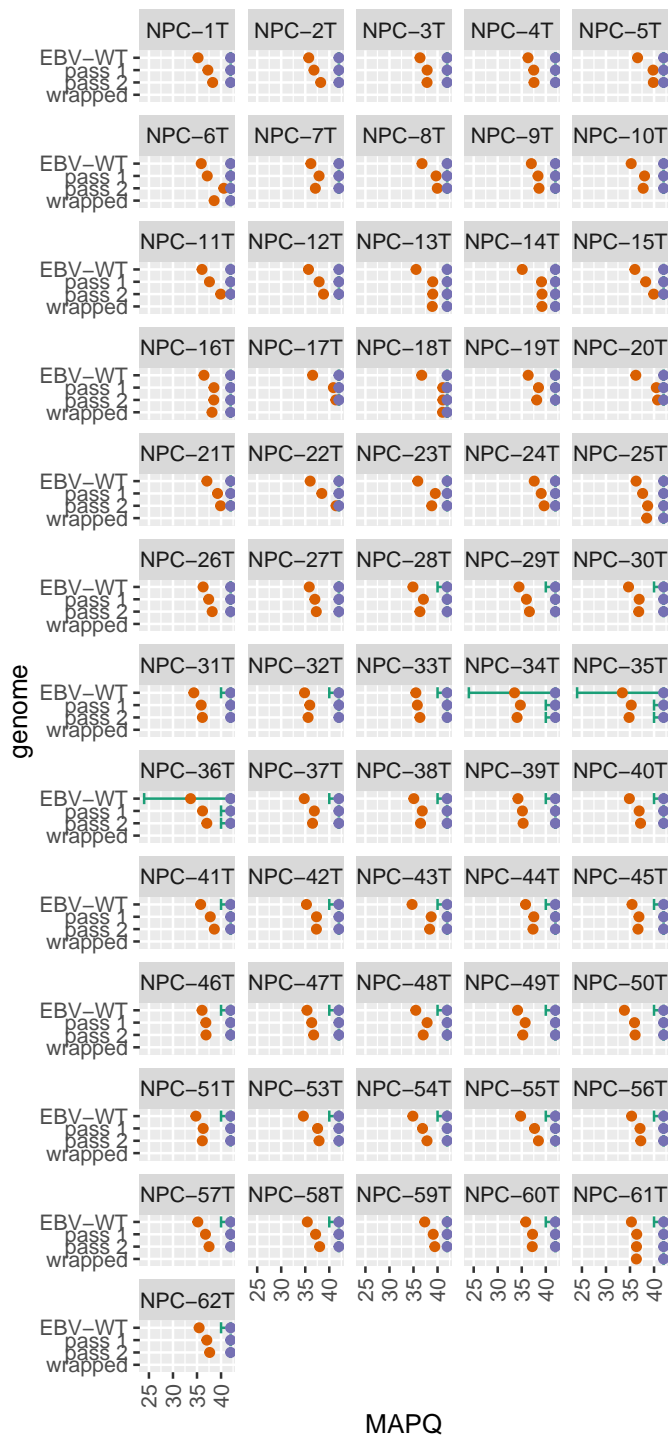

(b)

Supplement: Supplementary file 2 — Additional file 2 Supplementary Figure S2. Comparing the wild-type EBV genome and the reconstructed viral genomes in all 61 samples with two iterations of refinement. [file 12864_2022_8649_MOESM2_ESM.pdf]
