# Supplementary material for: Accurate reconstruction of viral genomes in human cells from short reads using iterative refinement
Source: BMC Genomics. 2022 Jun 6;23:422. doi: 10.1186/s12864-022-08649-8 (PMC9169298; doi:10.1186/s12864-022-08649-8)
Supplement: Supplementary file 4 — Additional file 4 Supplementary Table S1. Number of sequencing reads in each sample after different steps. The “raw”, “filtered”, “trimmed” and “EBV-WT” columns respectively show the number of millions of reads in the raw WGS data, after filtering human and mouse reads, after adapter trimming, and alignable to the EBV wild-type genome. [file 12864_2022_8649_MOESM4_ESM.pdf]

sample	read count (million)			
	raw	filtered	trimmed	EBV-WT
NPC-1T	2 721	33.53	27.82	1.699
NPC-2T	2 758	28.84	22.38	3.012
NPC-3T	2 762	34.07	26.69	2.090
NPC-4T	2 939	28.29	22.20	2.531
NPC-5T	2 737	30.66	23.79	1.629
NPC-6T	2 624	34.31	25.96	0.622 2
NPC-7T	2 809	49.72	34.77	4.914
NPC-8T	2 836	42.02	31.14	2.054
NPC-9T	2 839	41.39	30.21	5.776
NPC-10T	2 727	34.70	21.81	2.585
NPC-11T	2 771	32.82	27.30	0.969 0
NPC-12T	2 732	37.71	28.85	0.932 7
NPC-13T	2 715	29.37	23.24	0.986 7
NPC-14T	2 788	26.68	21.1	0.217 4
NPC-15T	2 756	25.03	19.21	2.017
NPC-16T	2 738	34.77	25.62	2.369
NPC-17T	2 663	34.91	24.89	0.765 9
NPC-18T	2 696	30.19	23.95	0.888 3
NPC-19T	2 769	30.91	22.72	0.923 3
NPC-20T	2 774	34.48	27.76	0.951 6
NPC-21T	2 664	28.22	23.10	1.146
NPC-22T	2 763	25.09	20.53	0.516 4
NPC-23T	3 007	37.00	31.22	1.461
NPC-24T	2 812	26.54	21.30	2.139
NPC-25T	2 667	29.11	23.11	1.398
NPC-26T	2 704	27.34	19.65	2.099
NPC-27T	2 843	39.18	30.86	5.369
NPC-28T	2 466	34.33	28.28	1.849
NPC-29T	2 481	30.57	24.21	2.260
NPC-30T	2 094	31.78	25.82	1.109
NPC-31T	2 496	39.33	33.58	1.415
NPC-32T	2 700	35.93	27.10	3.665
NPC-33T	2 478	28.69	24.79	6.525
NPC-34T	2 190	44.07	35.18	1.542
NPC-35T	2 147	41.49	32.90	3.558
NPC-36T	2 010	35.31	27.04	2.775
NPC-37T	2 015	33.53	27.30	0.955 1
NPC-38T	2 144	33.31	26.77	1.419
NPC-39T	2 263	37.47	29.31	0.449 5
NPC-40T	2 405	43.75	35.75	3.749
NPC-41T	2 384	36.14	29.57	1.290
NPC-42T	2 437	56.12	48.31	16.46
NPC-43T	2 641	78.96	69.08	5.740
NPC-44T	2 397	43.22	37.67	2.593
NPC-45T	2 397	43.42	37.91	1.067
NPC-46T	2 390	48.18	42.29	10.01
NPC-47T	2 185	35.03	30.88	2.004
NPC-48T	2 113	33.04	29.06	1.491
NPC-49T	2 100	51.23	42.83	2.874
NPC-50T	2 431	64.17	46.97	2.353
NPC-51T	1 981	45.34	37.98	2.004
NPC-53T	2 384	39.17	33.68	0.913 4
NPC-54T	2 322	39.35	33.38	0.732 5
NPC-55T	2 233	37.61	33.59	0.515 3
NPC-56T	2 181	37.31	33.09	2.240
NPC-57T	2 344	31.55	27.05	2.002
NPC-58T	2 273	29.34	25.11	1.428
NPC-59T	2 532	42.48	37.78	2.474
NPC-60T	2 619	42.08	37.49	6.801
NPC-61T	2 389	29.62	26.07	0.596 0
NPC-62T	2 458	32.85	28.96	3.222
