# Supplementary material for: Accurate reconstruction of viral genomes in human cells from short reads using iterative refinement
Source: BMC Genomics. 2022 Jun 6;23:422. doi: 10.1186/s12864-022-08649-8 (PMC9169298; doi:10.1186/s12864-022-08649-8)
Supplement: Supplementary file 3 — Additional file 3 Supplementary Figure S3. Alignment of the EBV genomes reconstructed with ASPIRE in NPC43 and C17 cells to the reference EBV genome sequence. [file 12864_2022_8649_MOESM3_ESM.pdf]

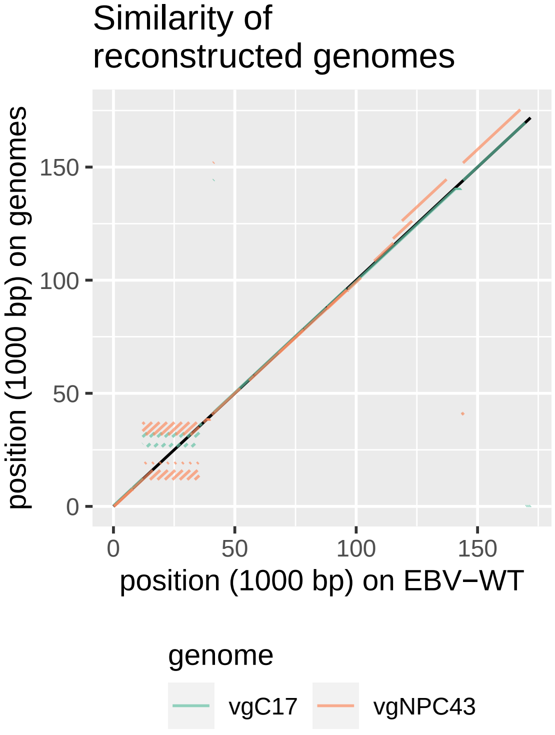
Similarity of
reconstructed genomes
position (1000 bp) on genomes
150
100
50
0
0
50
100
150
position (1000 bp) on EBV−WT
genome
vgC17
vgNPC43
